# Effect of *qGN4.1* QTL for Grain Number per Panicle in Genetic Backgrounds of Twelve Different Mega Varieties of Rice

**DOI:** 10.1186/s12284-017-0195-9

**Published:** 2018-01-22

**Authors:** Vijay Kumar Singh, Ranjith Kumar Ellur, Ashok Kumar Singh, M. Nagarajan, Brahma Deo Singh, Nagendra Kumar Singh

**Affiliations:** 1 ICAR-National Research Centre on Plant Biotechnology, Pusa Campus, New Delhi, 110012 India; 2 Division of Genetics, ICAR-Indian Agricultural Research Institute (ICAR-IARI), Pusa Campus, New Delhi, 110012 India; 3 ICAR-IARI-Rice Breeding and Genetics Research Centre, Aduthurai, Tamil Nadu 612101 India; 4 School of Biotechnology, Banaras Hindu University, Varanasi, 221005 India

**Keywords:** Marker-assisted backcross breeding (MABB), Grain number, *qGN4.1*, Rice, Near-isogenic lines (NILs), Yield

## Abstract

**Background:**

Rice is a major source of food, particularly for the growing Asian population; hence, the utilization of genes for enhancing its yield potential is important for ensuring food security. Earlier, we have mapped a major quantitative trait loci (QTL) for the grain number per panicle, *qGN4.1,* using biparental recombinant inbred line (RIL) populations involving a new plant type Indica rice genotype Pusa 1266. Later, three independent studies have confirmed the presence of a major QTL for spikelet number by three different names (*SPIKE, GPS* and *LSCHL4*) in the same chromosomal region, and have implicated the overexpression of *Nal1* gene as the causal factor for high spikelet number. However, the effect of *qGN4.1* in different rice genetic backgrounds and expression levels of the underlying candidate genes is not known.

**Results:**

Here, we report the effect of *qGN4.1* QTL in the genetic backgrounds of 12 different high-yielding mega varieties of rice, introgressed by marker assisted-backcross breeding (MABB) using two QTL positive markers for foreground selection and two QTL negative flanking markers for recombinant selection together with phenotypic selection for the recovery of recipient parent genetic background. Analysis of the performance of BC_2_F_3_ plants showed a significant increase in the average number of well-filled grains per panicle in all the backgrounds, ranging from 21.6 in CSR 30-*GN4.1* to 147.6 in Samba Mahsuri-*GN4.1*. Furthermore, *qGN4.1* caused a significant increase in flag leaf width and panicle branching in most backgrounds. We identified BC_3_F_3_ *qGN4.1* near-isogenic lines (NILs) with 92.0–98.0% similarity to the respective recipient parent by background analysis using a 50 K rice SNP genotyping chip. Three of the NILs, namely Pusa Basmati 1121-*GN4.1*, Samba Mahsuri*-GN4.1* and Swarna*-GN4.1*, showed a significant yield superiority to their recipient parents. Analysis of differential gene expression revealed that high grain number in these QTL-NILs was unlikely due to the overexpression of *Nal1* gene (LOC_Os04g52479). Instead, another tightly linked gene (LOC_Os04g52590) coding for a protein kinase domain-containing protein was consistently overexpressed in the high grain number NILs.

**Conclusion:**

We have successfully introgressed the *qGN4.1* QTL for high grain number per panicle into 12 different mega varieties of rice using marker-assisted backcross breeding. The advanced near-isogenic lines are promising for the development of even higher yielding versions of these high-yielding mega varieties of rice.

**Electronic supplementary material:**

The online version of this article (10.1186/s12284-017-0195-9) contains supplementary material, which is available to authorized users.

## Background

Food shortage is becoming a serious global problem as the rate of increase in world population exceeds the rate of increase in food production. In this regard, rice, which is one of the most important staple food crops feeding more than half of the human population worldwide, grown on the most productive irrigated land has achieved nearly maximum production with the current varieties. There is immense dependence on rice to satiate the food need of a large population and decrease the hunger index. With the completion of rice genome project, efforts are on to characterize the genes responsible for yield component traits such as the number of panicle-bearing tillers per plants, number of well-filled grains (spikelets) per panicle, and thousand grain weight with profound implications on yield improvement. Although the mechanisms that regulate each component trait are not yet fully understood, the genes and the available knowledge on the associated molecular markers offer a set of tools that can be combined to achieve higher grain-yielding varieties.

Grain yield is a complex trait controlled by several quantitative trait loci (QTLs), most of which have minor effects but some also have a major effect. Due to its complexity, different allelic combinations can give rise to similar phenotypes. It is still unclear whether a single QTL has the potential to enhance grain yield in diverse genetic backgrounds of elite rice varieties, though there is an example of *sd1* gene showing a consistent effect on grain yield enhancement of Indica rice varieties, leading to green revolution (Sasaki et al. 2002). Although many QTLs have been identified for the yield component traits (www.gramene.org/archive/QTL data), only a few of them have been evaluated for their impact on grain yield. Most of the QTLs for grain yield have been detected in crosses between Japonica and Indica cultivar groups because inter-cultivar group diversity is significantly higher than intra-cultivar group diversity. QTL-based marker-assisted breeding has been advocated for the transfer of beneficial genes into elite cultivars for food security (Takeda and Matsuoka 2008).

Many QTLs and genes affecting grain yield have been identified in rice during the last decade. A few examples are as follows: *Gn1a* and *APO1* for number of grains per panicle (Ashikari et al. 2005; Ikeda-Kawakatsu et al. 2009; Terao et al. 2010); *GS3*, *GW2*, and *qSW5* for grain size (Fan et al. 2006; Song et al. 2007; Shomura et al. 2008); *DEP1* and *WFP* for panicle architecture (Huang et al. 2009; Miura et al. 2010); *SCM2* for strong culm (Ookawa et al. 2010); *Ghd7* for late heading and number of grains (Xue et al. 2008) and *NAL1* (*SPIKE*, *GPS* and *LSCHL4*) for plant architecture and photosynthesis rate (Fujita et al. 2013; Takai et al. 2013; Zhang et al. 2014). *DEP1, SCM2* and *APO1* genes have been found to enhance grain yield in Japonica rice backgrounds in field experiments (Terao et al. 2010; Huang et al. 2009; Ookawa et al. 2010). A QTL *qGN4.1* with major effect on grain number per panicle was stable across 3 years with high LOD scores of 13, 6.8 and 5.3, explaining 27%, 16% and 12% of phenotypic variation, respectively (Deshmukh et al. 2010). The QTL *qGN4.1* was first mapped on the long arm of rice chromosome 4 in two different recombinant inbred line (RIL) populations (Pusa 1266/Pusa Basmati 1 and Pusa 1266/Jaya) derived from a new plant type (NPT) Indica rice genotype Pusa 1266 (Deshmukh et al. 2010; Marathi et al. 2012). This QTL is co-located with other important QTLs for the number of primary and secondary branches per panicle, number of tillers per plant, and flag leaf length and width, which may be due to either a tight genetic linkage or pleiotropic effects of the same gene on multiple traits (Deshmukh et al. 2010). No major gain in yield potential has been reported with the new plant type (NPT) breeding lines developed by IRRI due to poor grain filling and low biomass production. These drawbacks may be due to low crop growth rate during vegetative stage in the NPT lines as compared to Indica cultivars (Yamagishi et al. 1996), dense arrangement of spikelets on the panicle (Khush and Peng 1996), a limited number of large vascular bundles for assimilate transport, and source constraint due to early leaf senescence (Ladha et al. 1998). The introduction of high grain number trait from new plant type cultivars into recipient lines has also been initiated to broaden the genetic background of the NPT germplasm and refine the original ideotype design for increasing grain filling percentage and biomass production (Peng et al. 1999).

In the present study, we used marker-assisted backcross breeding (MABB) approach to introgress *qGN4.1* QTL for high grain number per panicle into genetic backgrounds of nine Indica and three Basmati rice varieties. A 50 K SNP genotyping chip was used for background selection in all the backcross-derived lines to ensure maximum recipient parent genome (RPG) recovery. The effect of *qGN4.1* QTL on grain yield in diverse genetic backgrounds was evaluated in field trials.

## Methods

### Plant Material

The donor parents (DP) used in this study, namely HG28 and HG67, were *qGN4.1* QTL-positive RILs derived from the cross between an NPT Indica rice genotype Pusa 1266 and Pusa Basmati 1 (Deshmukh et al. 2010). Recipient parents (RP) were all *qGN4.1* QTL-negative mega varieties of rice that are being cultivated in large acreage in India, namely Pusa Basmati 1121, Samba Mahsuri, Swarna, IR 64, MTU 1010, HUR 105, Sarjoo 52, Pusa 44, CSR 30, Ranjit, CR 1009, and Pusa Basmati 1. Pusa Basmati 1121, CSR 30 and Pusa Basmati 1 are Basmati cultivars, whereas the other RP varieties belong to Indica cultivar group. The seeds were obtained from the collection maintained at ICAR-NRCPB and Division of Genetics, ICAR-IARI, New Delhi.

### Plant DNA Extraction

The genomic DNA was isolated from the leaves of field-grown plants three to 4 weeks after transplanting. The leaf tissues from individual plants were frozen in liquid nitrogen, and DNA was extracted using the method of Murray and Thompson (1980). The quality and quantity of DNA were checked by electrophoresis in 0.8% agarose gel in 1× TBE buffer and comparison with known amounts of lambda DNA.

### Foreground and Recombinant Selection Using *qGN4.1* Flanking SSR Markers

**Fig. 1:**
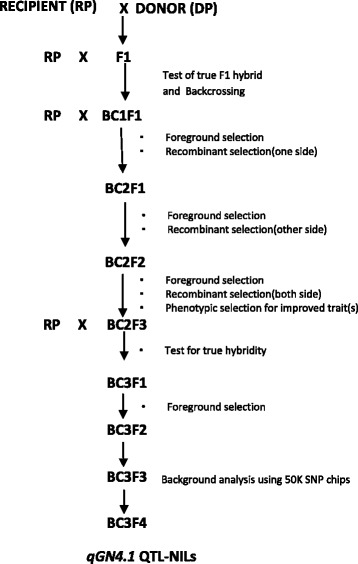
Crossing and selection scheme for the transfer of high grain number QTL *qGN4.1* in to popular high yielding mega varieties of rice

The *qGN4.1* QTL was transferred into 12 different mega varieties of rice using the previously described MABB strategy (Fig.1, Singh et al. 2015). In the first step, foreground selection for the presence of *qGN4.1* was performed using two QTL-positive markers (nkssr 04-11 and nkssr 04-19), which reduced the number of BC_n_F_1_ plants by 50%. In the second step, two QTL-negative recombinant selection markers (RM2441 and HVSSR 4-49) flanking the QTL-positive markers on both sides were used to identify lines with minimum linkage drag of the DP (Additional file 1: Table S6). The *qGN4.1* QTL has been narrowed down to a 360 kb interval between QTL-positive SSR markers nkssr 04-11 and nkssr 04-19 (Deshmukh et al. 2010, Sharma 2013). The PCR reaction consisted of 1.0 μl of 10× reaction buffer, 0.15 μl of 10 mM dNTPs (133 μM), 1.0 μl each of forward and reverse primers (10 pmol), and 2.0 μl of template genomic DNA (20-30 ng), 0.10 μl of Taq DNA polymerase in a final reaction volume of 10 μl. The PCR was carried out using Bio-Rad thermal cycler with an initial denaturation for 5 min at 94 °C, followed by 35 cycles for denaturation at 94 °C for 30 s, annealing at 55 °C for 30 s, extension at 72 °C for 1 min, and a final extension at 72 °C for 10 min. The PCR amplicons of nkssr 04-11, nkssr 04-19 and RM2441 were resolved by electrophoresis in 3.5–4% Metaphor™ Agarose gel and visualized on UV transilluminator (Gel Doc™ XR + Imager, Bio-Rad Laboratories Inc., U.S.A). However, because of the small size difference between donor and recipient alleles of HvSSR 04-49, it was analyzed by capillary electrophoresis of *fam* dye-labeled PCR products using ABI 3730 XL Genetic Analyzer.

### Analysis of Recipient Parent Genome Recovery Using 50 K SNP Chip

In early generations of backcrossing, the background selection for RPG recovery was performed on the basis of morphological similarity of the backcross-derived lines with the RP (Singh et al. 2015). However, at BC_3_F_3_ stage, marker-assisted background analysis was performed on multiple QTL-near-isogenic lines (NILs) selected for maximum phenotypic similarity with the RP. For genotypic background selection, the short-listed QTL-NILs were compared with respective RPs using the Affymetrix 50 K Axiom® 2.0 SNP chip ‘OsSNPnks’ on GeneTitan® instrument (Singh et al. 2015). High-quality DNA with OD_260/280_ and OD_260/230_ in the range of 1.8–2.0 was used for SNP genotyping. Genomic DNA amplification, fragmentation, chip hybridization, washing, single-base extension through DNA ligation, and signal amplification was carried out according to Affymetrix Axiom® 2.0 Assay Manual Target Prep Protocol. Approximately 0.75–1.00 μg genomic DNA was labeled overnight at 25 °C using three volumes of the BioPrime DNA labeling reaction. The labeled DNA was ethanol precipitated, resuspended in 40 μl H_2_O and then added to Affymetrix SNP 6.0 hybridization cocktail. Staining, washing, and scanning were performed using GeneTitan integrated platform (http://www.affymetrix.com). SNP genotypes were called using the Affymetrix Genotyping Console™ v4.1 (AGC) software package. SNPs with low call rates across all samples were removed from the dataset and high-performing SNPs with a development quality check (DQC) score of >0.85, and call rates of >95.0% were used for further analyses (Singh et al. 2015). Graphical representation of 50 K SNP genotyping based RPG recovery was done using Phenogram software from Ritchie lab, Penn. State University, Pennsylvania, USA (http://visualization.ritchielab.psu.edu/phenograms/plot). The RPG similarity based on SNP markers was calculated using the formula, RPG (%) = (R + 1/2H) × 100/P, where R = number of markers homozygous for RP allele, H = number of heterozygous markers, P = total number of SNP markers used for background selection (Ellur et al. 2015).

### Measurement and Analysis of Field Phenotyping Data

Phenotypic traits were evaluated on BC_2_F_3_ plants using augmented field design. All the 12 RPs and six selected NILs for each recipient were planted across five blocks without replication with four checks repeated in each block. Measurements were taken on five plants for each entry for the following ten traits. Plant height was measured in cm from the base of the main stem to the tip of the primary panicle of the plant. The total number of tillers was recorded as both productive and unproductive tillers in a single plant. Productive tillers per plant referred to the number of tillers-bearing panicle at harvest. Flag leaf length was measured as the length of leaf-bearing primary panicle from leaf base to the tip. Flag leaf width was the maximum width of the leaf-bearing primary panicle. Panicle length was the length of primary panicles measured from the base of peduncle to the tip excluding awns. Primary branches per panicle referred to the total number of branches coming out directly from the peduncle. Secondary branches per panicle referred to the total number of branches coming out from the primary braches of the panicle. Spikelet fertility was measured as the number of well-filled grains/total number of grains per panicle × 100. The total number of grains per panicles was measured as the total number of florets (spikelets) present in the primary panicle. All the NILs and their respective RP varieties were planted in two-meter rows with row-to-row spacing of 30 cm and plant-to-plant spacing of 20 cm. The best five BC_2_F_3_ plants showing the maximum phenotypic similarity with the RP were used for measuring plant height, flag leaf length, flag leaf width, tiller number and number of grain per panicle. Pollen from one of the plants most similar to RP was used for backcrossing to produce BC_3_ seeds.

Field evaluation of yield and morphological traits was carried out in the experimental fields of Rice Breeding and Genetics Centre, Aduthurai (Tamil Nadu) and Indian Agricultural Research Institute, New Delhi. Evaluation of yield-associated traits was done on NILs, their RPs and four checks using an augmented randomized complete block design (RCBD) with five blocks, each having a maximum of 21 entries including four checks. The data for each character were analyzed as per the procedure of augmented RCBD using the Statistical Package for Augmented Designs. The treatment sum of squares was partitioned into the sum of squares among NILs, among checks and between NILs and checks. The adjusted means of NILs and checks were obtained. For making all possible pair-wise treatment comparisons, critical difference was calculated at 5% level of significance (*P* = 0.05) for each of the four comparisons, namely between checks, between NILs and checks, between NILs of the same block, and between NILs of different blocks (Federer 1956, 1961; Parsad and Gupta 2000). The backcross-derived lines along with the respective RPs were evaluated for yield (kg/ha) at BC_3_F_4_ generation in an RCBD with two replications.

### RNA Extraction and qRT-PCR Analysis of Candidate Gene Expression

Total RNA was extracted from panicle primordia measuring 2–3 cm in length using Promega’s SV Total RNA Isolation System. The concentration of each RNA sample was measured using NanoDrop ND-1000 spectrophotometer (NanoDrop Technologies). Only the RNA samples with 260/280 ratio between 1.9 and 2.1 and 260/230 ratio greater than 2.0 were used for the analysis. First-strand cDNA was synthesized from 6 μg of total RNA using oligo dT as a primer and reverse transcriptase mix (AffinityScript QPCR cDNA Synthesis Kit; Agilent Technologies) in a 20 μl reaction volume. The PCR mixture contained 2 μl of template cDNA, 5 μl of 2× SYBR Green qPCR Master Mix (Agilent Technologies), 0.4 μl of gene-specific forward and reverse primers, 0.15 μl of ROX reference dye1, and 2.05 μl of nuclease-free H_2_O. Gene expression was normalized against eEF1a as an internal control. Control PCR reaction with no template was also performed for each primer pair. The RT-PCR was performed using Agilent Technologies Stratagene, Mx3005P Detection System and software. The PCR amplification condition included one cycle of 95 °C (3 min), followed by 40 cycles of denaturation at 95 °C (30 s), 60 °C (20 s), and 72 °C (20 s), interrupted by the dissociation curve with denaturation at 95 °C (1 min), cooling at 55 °C (30 s) and gradually heating up to 95 °C (30 s) in 96-well optical reaction plate. The amplicon purity was determined when a single melting peak was reached. Two biological replicates for each sample and three technical replicates for each biological replicate were used for RT-PCR analysis. The rice eEF1a gene was used as internal control (primer pair 5′TTTCACTCTTGGAGTGAAGCAGAT3′ and 5′GACTTCCTTCACGATTTCATCGTAA3′). Gene-specific primers were designed using the online PrimerQuest tool of IDT-Integrated DNA Technologies (https://eu.idtdna.com/PrimerQuest/Home/Index) using full-length cDNA sequences of the three candidate genes in the QTL interval: (i) *Nal1* (LOC_Os04g52479); (ii) Retrotransposon gene (LOC_Os04g52540), and (iii) Protein kinase domain containing protein gene (LOC_Os04g52590), (http://rice.plantbiology.msu.edu/cgi-bin/gbrowse/rice/#search). Primers for qRT-PCR expression analysis were: 5′GGTGTTTGGTGTGATGTTGATG3′ and 5′CCTGAGAGCCTGAACCAATAC3′ for LOC_Os04g52479 (product size 136 bp); 5′TCAGGTCTTCGCATTGGCACAC3′ and 5′GTACCGTTGAGCGCATTGCTTC3’ for LOC_Os04g52540 (product size 105 bp); and 5′GCAGCTCTCTCAGTCATCTAATC3′ and 5′TCTCAAGGCAGCAACCATAC3′ for LOC_Os04g52590 (product size 113 bp). Relative expression levels of the three genes were assayed on a real-time PCR system (Agilent Technologies Stratagene, Mx3005P). The relative gene expression of the target gene was calculated using the equation: Exp = 2^-ΔCt^, where Δ*Ct* = *Ct*_*t*arg*etgene*_ − *Ct*_*eEF1a*_ (Livak and Schmittgen 2001).

## Results and Discussion

### *qGN4.1* QTL-NILs Developed by Marker-Assisted Backcross Breeding

**Fig. 2:**
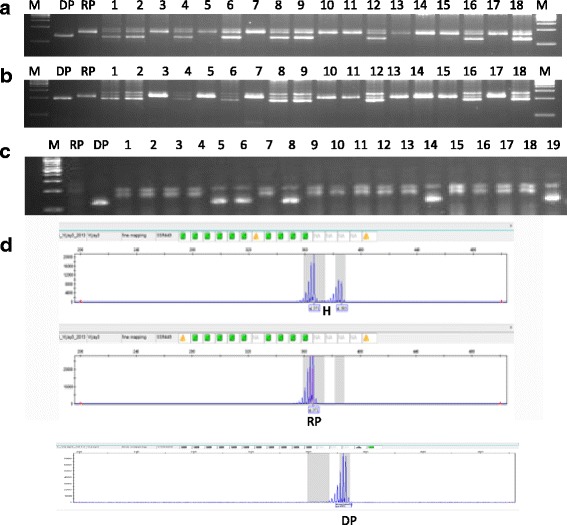
Foreground (**a**, **b**) and recombinant (**c**, **d**) selection in backcross F_1_ lines of variety Ranjit with linked SSR markers **a**. nkssr 04-11 **b**. nkssr 04-19. **c**. RM2441 using 3.5-4% metaphor agarose gel electrophoresis, and **d** HVSSR 04-49 by capillary electrophoresis. M = DNA size markers, RP = recipient parent, DP = donor parent, H = F1 heterozygote, 1-18 BC1F1 plants. The bands marked RP and DP on the gel picutre are the main bands

We transferred *qGN4.1*, a major QTL for grain number per panicle, into 12 different mega varieties of rice by marker-assisted backcross breeding (Fig. 1). In addition to marker-assisted selection using two tightly linked QTL-positive foreground selection markers and two flanking QTL-negative recombinant selection markers, the backcross progenies were also selected for phenotypic similarities with the respective RP at each filial generation. The background selection was initially restricted to this visual screening of phenotypic traits for similarity with the recipient parent. The backcross progeny was selected for the high grain number donor parent (DP) alleles of the foreground selection markers nkssr 04-11 and nkssr 04-19 tightly linked to *qGN4.1*, and the recombinant selection was for the presence of RP alleles of QTL flanking markers RM 2441 and HvSSR 04-49 (Fig. 2, Additional file 1: Table S6). Details of the number of backcross progenies planted in each generation for all the 12 RP varieties and the number of plants selected positive for both foreground and recombinant selection markers for each cross are shown in Additional file 6: Table S1. Out of the 12 crosses, both side recombinants were recovered in BC_2_F_1_ generation for ten crosses, except for HUR 105 and MTU 1010 for which both side recombinants were selected in the BC_2_F_3_ and BC_3_F_1_ generations, respectively (Additional file 2: Table S1). The selected lines recombinant on both sides of the QTL was taken further to the next generation for selection of lines with maximum visual similarity to the respective RP. Finally, six lines were selected for each RP variety for the presence of DP alleles of QTL-positive foreground selection markers, both side RP parent alleles of the QTL-negative recombinant selection marker and phenotypic similarity with RP for background RPG recovery and designated RP-*qGN4.1-1* to RP-*qGN4.1-6* (Additional file 7: Table S2).

### Phenotypic Characteristics and Yield Performance of *qGN4.1* QTL-NILs at BC_2_F_3_

**Fig. 3:**
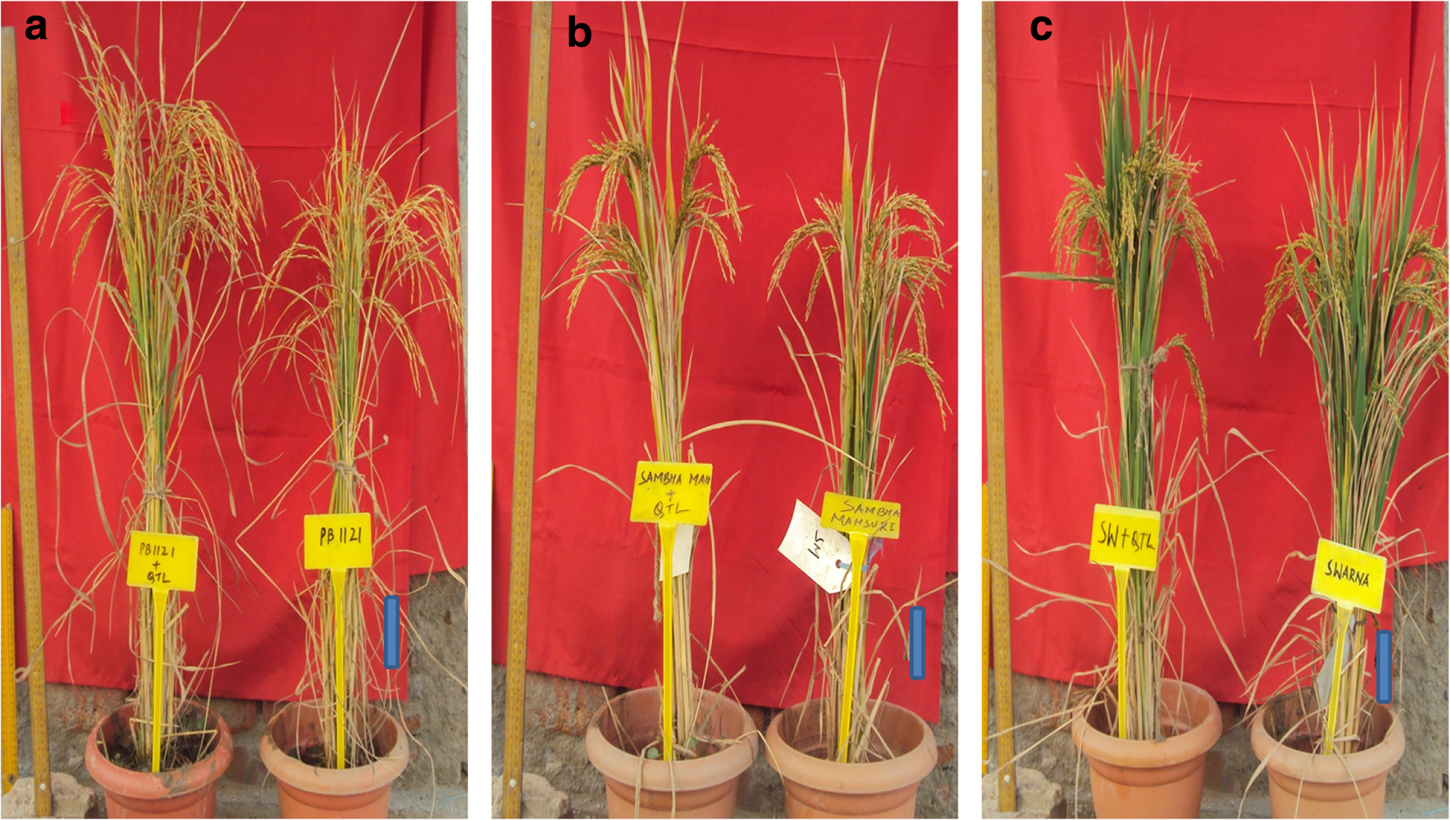
Plant architecture of *qGN4.1* QTL-NILs (left side) of rice as compared to their recipient parents (right side): **a** Pusa Basmati 1121(PB1121) **b** Samba Mahsuri **c** Swarna (scale bars: 10 cm)

**Fig. 4:**
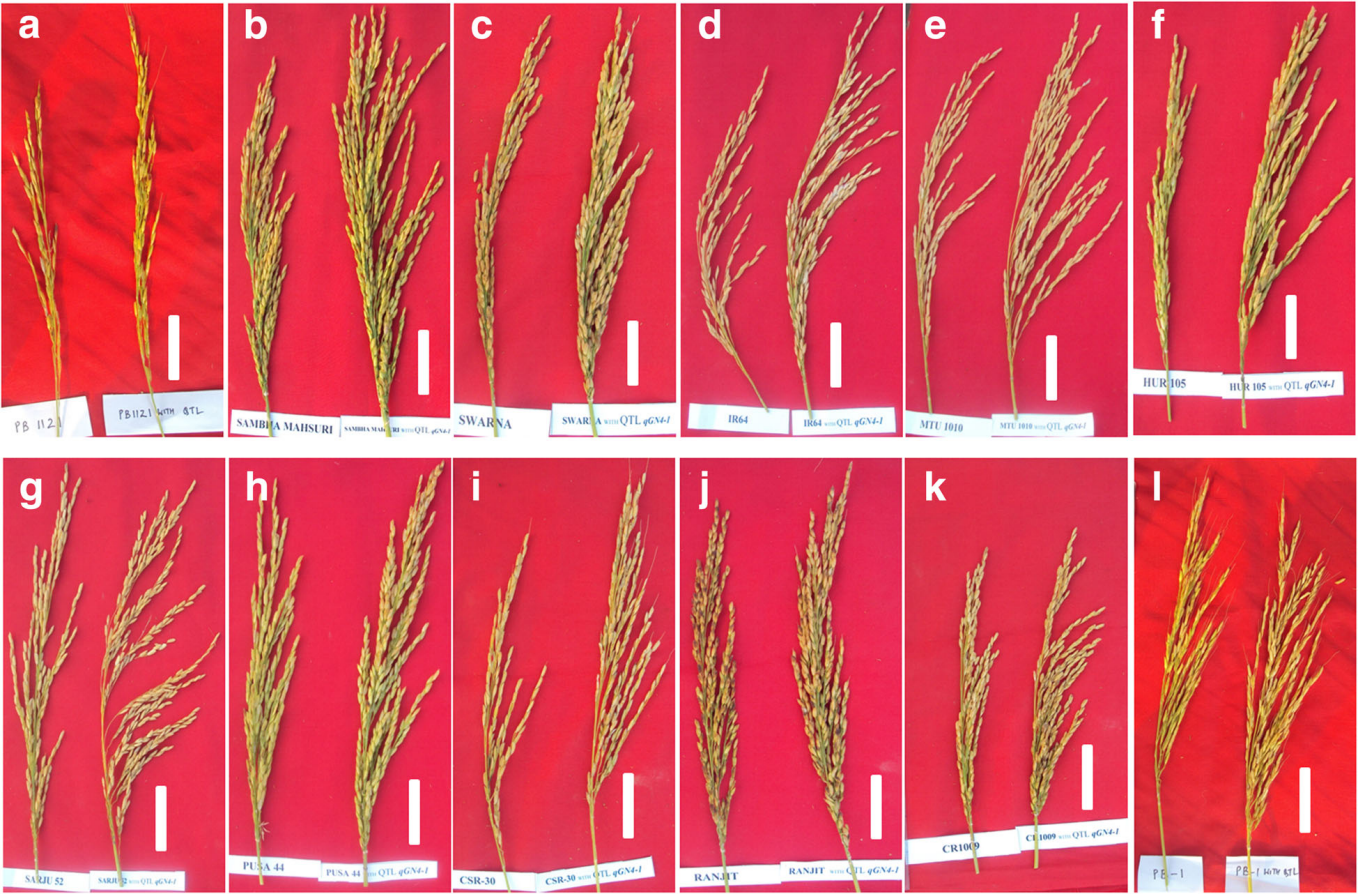
Panicle structure of *qGN4.1* QTL-NILs (right side) along with respective recipient parents (left side): **a** Pusa Basmati 1121 (PB 1121) **b** Samba Mahsuri, **c** Swarna **d** IR 64 **e** MTU 1010 **f** HUR 105 **g** Sarjoo 52 **h** Pusa 44 **i** CSR 30 **j** Ranjit **k** CR 1009 **l** Pusa Basmati 1(PB 1), Scale bars- 5 cm

Six selected *qGN4.1* QTL-NILs each for the 12 different RP genetic backgrounds at BC_2_F_3_ generation were planted along with their respective RPs for evaluation in an augmented block design (Additional file 3: Table S2). Phenotypic data were recorded for ten traits, including plant height (PH), total number of tillers (TT), number of productive tillers (PT), flag leaf length (FLL), flag leaf width (FLW), panicle length (PL), number of primary branches per panicle (PB), number of secondary branches per panicle (SB), total number of grains per panicle (TG), number of well-filled grains per panicle (FG) and spikelet fertility (SF) (Table 1). The mean values of data from five plants for each QTL-NIL were adjusted based on the performance of the check varieties in that block, resulting in adjusted means. For each of the 12 RP varietal backgrounds plant morphology of the NILs was similar to the respective RP because of good RPG background recovery in the NILs (Fig. 3; Additional file 4: Figure S1; Additional file 5: Figure S2; Additional file 6: Figure S3). For the ten phenotypic traits compared there was no statistically significant difference between NILs and their respective parents for panicle length, but plant height increased significantly in case of PB 1121-*qGN4.1*, IR 64-*qGN4.1* and MTU 1010-*qGN4.1*. The total number of tillers decreased significantly in PB 1121-*qGN4.1*, IR 64- *qGN4.1*, HUR 105-*qGN4.1* and Sarjoo 52-*qGN4.1* but the number of productive tillers decreased significantly only in PB 1121-*qGN4.1* and Sarjoo 52-*qGN4.1*. Flag leaf length increased significantly in HUR 105-*qGN4.1*, Sarjoo 52-*qGN4.1* and PB 1-*qGN4.1* while flag leaf width increased significantly in PB 1121-*qGN4.1*, Samba Mahsuri-*qGN4.1*, Swarna-*qGN4.1*, Pusa 44-*qGN4.1*, Ranjit-*qGN4.1*, and Pusa Basmati 1-*qGN4.1* (Table 1). The number of primary branches per panicle increased significantly in PB1121-*qGN4.1*, Samba Mahsuri-*qGN4.*1, Swarna-*qGN4.1*, IR64-*qGN4.1*, CSR30-*qGN4.1*, Ranjit-*qGN4.1* and PB1-*qGN4.1* while number of secondary branches per panicle increased significantly in PB1121-*qGN4.1*, Samba Mahsuri-*qGN4.1*, IR 64-*qGN4.1* MTU1010-*qGN4.1*, and Ranjit-*qGN4.1*. The total number of grains (spikelets) and the number of well-filled grains per panicle both increased significantly in the QTL-NILs of all 12 RP genetic backgrounds. Panicle length, number of primary and secondary branches per panicle, total number of grains per panicle and spikelet fertility directly influence the number of well-filled grains per panicle, which has a direct relevance to enhancing of yield potential (Fig. 4). The increase in number of well-filled grains per panicle ranged from 21.6 in CSR 30 to 147.6 in Samba Mahsuri (Table 1).

**Table 1:** Adjusted Means for Different Morphological Traits for the *qGN4.1* QTL-NILs Compared With Respective Recipient Parents in BC_2_F_3_ Generation

Genotype	PH	TT	PT	FLL	FLW	PL	PB	SB	TG	FG	SF
PB1121	101.7	20.8	16.6	27.2	1.0	21.8	10.1	37.2	99.5	87.5	87.9
PB1121 + *qGN4.1*	117.1*	11.1*	9.3*	29.1	1.4*	25.3	12.2*	55.2*	128.7*	117.5*	91.3
Samba Mahsuri	84.5	12.6	9.3	25.9	1.3	17.6	11.5	55.2	284.1	235.9	83.0
SM + *qGN4.1*	87.5	8.5	7.1	30.3	2.3*	20.1	14.3*	68.1*	409.7*	383.5*	93.6*
Swarna	89.7	12.9	9.5	23.8	1.0	21.2	12.9	56.7	197.9	174.5	88.2
SW + *qGN4.1*	92.9	11.4	9.4	31.6	1.6*	24.7	14.9*	59.3	221.1*	208.1*	94.1*
IR 64	85.1	16	10.6	32.5	1.2	21.7	10.1	37.8	158.4	142.1	89.7
IR64 + *qGN4.1*	106.3*	9.9*	8.4	33.4	1.3	23.2	11.9*	54.5*	211.8*	197.7*	93.3
MTU1010	80.9	11.6	8.8	31.5	1.4	25.1	12.1	46.8	180.2	153.5	85.2
MTU + *qGN4.1*	98.9*	11.1	9.8	24.9	1.5	20.1	12.9	66.8*	210.9*	200.3*	95.0*
HUR 105	90.8	21	15.9	23.4	1.5	20.8	12.8	54.9	185.3	165.2	89.2
HUR + *qGN4.1*	93.2	15.6*	14.5	31.6*	1.2	23.7	13.4	60.3	208.5*	190.0*	91.1
Sarjoo 52	79	18.4	15.3	23.8	1.3	22.1	12.8	59.6	199.9	178.2	89.1
Sarjoo52 + *qGN4.1*	84.5	10.4*	9.6*	33.4*	1.5	24.3	13.9	69.1	239.7*	218.2*	91.0
PUSA 44	84.4	9.6	6.9	31.3	1.2	24.9	12.6	57.9	183.5	162.6	88.6
PUSA44 + *qGN4.1*	91.8	7.0	5.8	33.8	1.5*	25.8	13.0	61.6	226.7*	208.8*	92.1
CSR 30	105.2	14.7	10.4	26.0	1.0	25.1	10.4	31.5	121.4	105.1	86.6
CSR30 + *qGN4.1*	115.6	12.5	11.8	27.7	1.1	26.8	12.6*	36.5	134.4*	126.7*	94.3*
Ranjit	121.6	13.4	9.0	28.1	1.8	23.2	12	51.9	215.8	204.9	94.9
Ranjit + *qGN4.1*	125.8	11.1	9.7	28.5	2.2*	25.7	12.7	67.5*	317.6*	303.7*	95.6
CR 1009	93.1	15.5	10.9	21.2	1.3	19.4	12.1	56.7	175.1	152.0	86.8
CR1009 + *qGN4.1*	99.5	12.9	11.1	24.9	1.4	23.7	13.3*	60.5	215.1*	199.6*	92.8*
Pusa Basmati 1	96.5	12.2	10.4	22.8	0.6	23.7	11.7	52.6	154.5	137.5	89.0
PB1 + *qGN4.1*	101.6	11.6	10.3	29.4*	1.0*	20.3	14.5*	57.9	198.1*	180.3*	91.0
CD(0.05)	14.5	5.2	5.0	6.0	0.3	4.5	1.5	10.0	11.7	7.4	5.0

*PH* plant height in cm, *TT* total tillers, *PT* productive tillers, *FLL* flag leaf length in cm, *FLW* flag leaf width in cm, *PL* panicle length in cm, *PB* primary branches per anicle, *SB* secondary branches per panicle, *SF* percent spikelet fertility, *FG* filled grain per panicle, *TG* total grain number per panicle, *SM* Samba Mahsuri, *SW* Swarna. *Significantly from respective recipient parent (*P* > 0.05)

Plant height increased significantly in PB 1121, IR 64 and MTU 1010 genetic backgrounds leading to 15.4-21.2 cm taller plants with higher biomass accumulation without causing any lodging problem (Fig.3; Additional file 4: Figure S1; Additional file 5: Figure S2; Additional file 6: Figure S3). The total number of tillers decreased in the *qGN4.1* QTL*-*NILs at the cost of unproductive tillers, a typical characteristic of the new plant type donor variety. The number of productive tillers decreased significantly only in the Pusa 1121 and IR 64 background. Thus, there were similar numbers of healthy and sturdy productive tillers in most of the *qGN4.1* QTL-NILs as in the respective RPs. Comparison of the original new plant type (NPT) lines with the highest yielding Indica rice cultivars has shown that the original NPT lines had significantly lower tillering capacity (Peng et al. 1994). Sufficient tillering capacity is needed for biomass production to improve compensation when tillers are lost due to insect damage or other stresses during vegetative stage (Peng et al. 1999). Primary branches, secondary branches and the total number of filled grains per panicle are important parameters which would directly affect the grain yield of rice. All of these characteristics were directly correlated to each other leading to a larger panicle size (Fig. 4). Larger panicle size of *qGN4.1* QTL-NILs without significant change in panicle length was because of increase in panicle branching without more compact arrangement of spikelets which may result in poor grain filling. It is very useful that *qGN4.1* QTL-NILs in Sambha Mahsuri, Swarna, MTU 1010, Ranjit and CR 1009 backgrounds have significant improvement in percent spikelet fertility. The spikelet fertility was enhanced by as much as 10.3% in Samba Mahsuiri-*qGN4.1*, which might help develop an even higher yielding version of this popular mega variety of rice. Thus, this study clearly shows that introgression *qGN4.1* QTL significantly increased grain numbers per panicle in all the 12 QTL-NILs as compared to the respective RP without any reduction in spikelet fertility.

The QTL *qGN4.1* for grain number per panicle was first identified in our laboratory in an Indica/Indica RIL population of Pusa 1266/Pusa Basmati 1, including eight differentially expressed candidate genes in the QTL interval (Deshmukh et al. 2010). Three subsequent studies have also identified a QTL for high grain (spikelet) number in the same region of rice chromosome 4 but with different QTL names, viz. *SPIKE*, *GPS*, *LSCHL4* using Indica/Japanica crosses (Fujita et al. 2013; Takai et al. 2013; Zhang et al. 2014). The latter three studies have implicated the overexpression of previously described *NAL1* gene for narrow leaves mutation for the QTL effect (Qi et al. 2008). Here, we introgressed the *qGN4.1* QTL in the genetic backgrounds of 12 different Basmati and Indica rice cultivars to see its effect on different yield parameters. Thus, we can presume that *NAL1* allele could also be involved in enhancing flag leaf area in the *qGN4.1* QTL-NILs for enhanced photosynthetic efficiency, and grain number per panicle traits, but as shown below, the expression of *NAL1* gene was down-regulated in the high grain number lines in our material. The present study employed a MABB approach to demonstrate a consistent effect of *qGN4.1* QTL in diverse genetic backgrounds. We believe that *qGN4.1* QTL-NILs in the genetic backgrounds of different Indica and Basmati cultivars have improved photosynthetic efficiency through coordination of leaf morphological and physiological traits, which has great potential for use in breeding for higher-yielding rice varieties.

### Recipient Parent Genome Recovery and Yield Performance of the *qGN4.1* QTL-NILs

RPG recovery of the *qGN4.1* QTL-NILs was analyzed by comparing the percentage of marker allele similarity with the respective RP at BC_3_F_3_ generation using a 50 K SNP genotyping chip (Singh et al. 2015). We analyzed the six best QTL-NILs each for six RP varieties, namely Samba Mahsuri, Swarna, MTU 1010, Sarjoo 52, PUSA 44 and Pusa Basmati 1 (PB 1), and two best NILs each for the remaining six varieties, namely Pusa Basmati 1121, IR 64, HUR 105, CSR 30, Ranjit and CR 1009 due to resource limitations (Additional file 7: Table S3). During early generations of backcrossing until BC_3_F_2_ plants were selected for RPG recovery phenotypically, more attention was paid to foreground selection for the presence of donor QTL allele using QTL-positive markers and recombinant selection for the presence RP alleles using QTL-negative markers to minimize the linkage drag on the carrier chromosome. The QTL-NILs with the highest overall RPG recoveries for the 12 recipient rice varieties were Pusa Basmati 1121 (97.6%), Samba Mahsuri (96.5%), Swarna (93.8%), IR 64 (98.0%), MTU 1010 (93.8%), HUR 105 (96.5%), Sarjoo 52 (92.0%), Pusa 44 (94.3%), CSR 30 (97.4%), Ranjit (97.7%), CR 1009 (96.0%), and Pusa Basmati 1 (93.4%). RPG recoveries for QTL *qGN4.1* career chromosome 4 in these lines were: 94.6%, 93.1%, 89.6%, 95.4%, 90.6%, 93.3%, 78.6%, 91.3%, 85.8%, 91.3%, 90.3% and 91.5%, respectively (Table 2), which was significantly lower than the overall RPG recovery due to linkage drag of the donor genome in the QTL region. The recipient genome was almost fully recovered in the selected NILs at BC_3_F_4_ generation, and these lines were used for evaluating the effect of *qGN4.1* QTL on yield performance in different genetic backgrounds. Although the selected NILs still have 2-8% genomic content from the DP, it can be reduced further by additional backcrossing if needed.

**Table 2:** Percentage Genome Similarity of The Best *qGN4.1* QTL-NILs With Recipient Parent at BC3F2 Generation in the Genetic Background of Twelve Different Mega Varieties of Rice Analysed Using OsSNPnks SNP Genotyping Chip

Sr. no.	Recipient variety	Rice chromosome												Overall similarity
		1	2	3	4	5	6	7	8	9	10	11	12	
1.	PB 1121	99.1	95.0	98.5	94.6	97.1	98.8	95.8	98.8	95.3	98.2	98.6	97.2	97.6
2.	Samba Mahsuri	96.6	97.0	96.1	93.1	96.7	98.0	99.6	96.0	96.9	96.7	95.0	95.7	96.5
3.	Swarna	95.5	93.4	93.3	89.6	94.4	97.1	99.4	93.9	92.2	91.5	86.1	92.1	93.8
4.	IR 64	98.8	96.2	98.9	95.4	98.9	99.4	98.1	94.5	99.3	96.1	99.3	98.0	98.0
5.	MTU 1010	95.6	94.7	93.0	90.6	94.7	93.5	96.8	92.9	94.5	91.3	91.7	92.2	93.8
6.	HUR105	91.4	96.3	97.9	93.3	97.9	92.7	98.5	98.2	97.9	97.6	97.6	97.5	96.5
7.	Sarjoo 52	95.6	90.4	90.3	78.6	93.8	93.9	98.1	93.2	89.5	90.9	93.5	93.9	92.0
8.	Pusa 44	93.0	95.1	93.7	91.3	95.0	96.8	99.5	95.3	95.1	92.5	91.4	93.3	94.3
9.	CSR 30	99.6	95.9	97.8	85.8	98.5	99.6	96.9	92.6	99.6	99.4	99.3	99.2	97.4
10	Ranjit	97.9	99.2	94.9	91.3	99.5	99.5	99.4	99.5	98.7	93.9	99.2	99.6	97.7
11	CR1009	94.5	99.5	97.9	90.3	99.0	94.1	99.4	89.6	92.7	98.8	99.3	94.9	96.0
12	PB 1	97.2	94.2	92.1	91.5	95.3	96.3	97.1	95.2	89.2	89.2	90.1	93.3	93.4
	Average	96.2	95.6	95.4	90.5	96.7	96.6	98.2	95.0	95.1	94.7	95.1	95.6	95.5

**Fig. 5:**
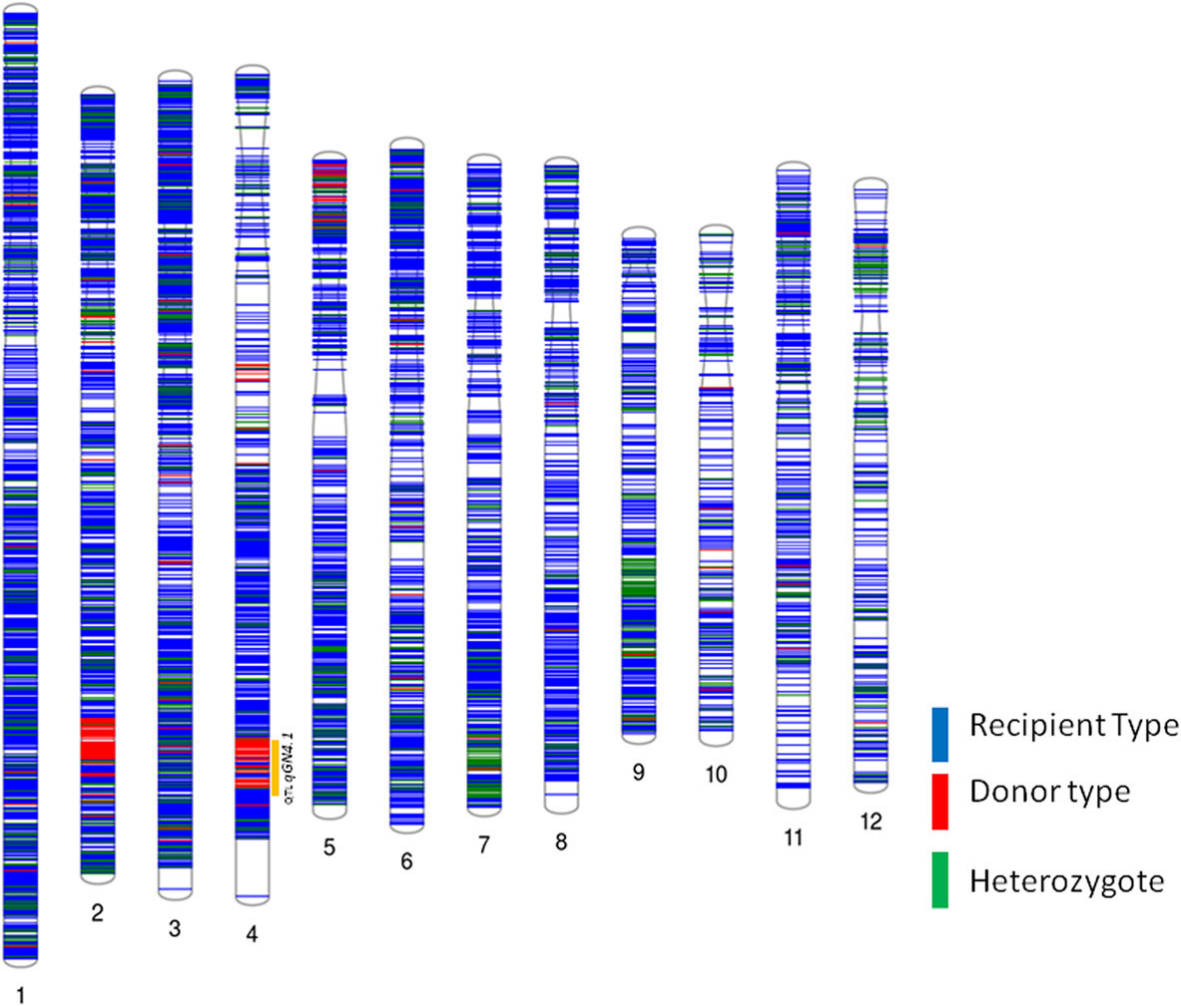
Graphical representation of RPG similarity of high grain number QTL-NIL PB 1121- *qGN4.1* with the PB 1121 using 50 K SNP

The RPG recovery was variable, but DP alleles were commonly present in the *qGN4.1* region of chromosome 4 in all the QTL-NILs as visualized by graphical genotyping (Fig. 5; Additional file 8: Figure S4; Additional file 9: Figure S5; Additional file 10: Table S5). A similar pattern of increased grain number per panicle and related traits across all the 12 RP backgrounds validate the effect of *qGN4.1* QTL on the grain number per panicle. Graphical representation of the high-resolution SNP genotyping data of QTL-NILs revealed the substantial size of DP chromosome segments introgressed at the *qGN4.1* QTL region on chromosome 4, viz. Pusa Basmati 1121 (2.2 Mb), Samba Mahsuri (2.7 Mb), Swarna (4.1 Mb), IR 64 (2.4 Mb), MTU 1010 (3.4 Mb), HUR 105 (4.2 Mb), Sarjoo 52 (1.2 Mb), Pusa 44 (2.5 Mb), CSR 30 (2.8 Mb), Ranjit (4.3 Mb), CR 1009 (2.3 Mb), and Pusa Basmati 1 (2.8 Mb) despite conscious efforts to reduce the linkage drag by recombinant selection using QTL-negative flanking markers (Table 2). In addition to the consistent presence of 1.2-4.3 Mb of DP chromosome segment in the QTL region, the QTL NILs also showed other random large donor chromosome segments (Additional file 11: Table S4). For example, PB 1121-*qGN4.1* carries 2.6 Mb, 0.7 Mb and 2.5 Mb segments of DP on chromosomes 2, 4 and 5, respectively (Fig. 5, Additional file 12: Table S7). Samba Mahsuri-*qGN4.1* has 48 DP segments of >0.2 Mb on chromosomes 1, 2, 8, 11 and 12; Swarna-*qGN4.1* has 75 DP segments of >0.2 Mb on chromosomes 1, 3, 5, 11 and 12; IR 64-*qGN4.1* has five large DP segments on chromosomes 1 (0.4 Mb), 2 (1.8 Mb), 7 (0.6 Mb), 8 (3.7 Mb) and 12 (2.2 Mb); MTU 1010-*qGN4.1* has 163 DP segments of >0.2 Mb through out the genome except chromosomes 7; HUR 105-*qGN4.1* has 43 DP segments of >0.2 Mb on chromosome 1, 2, 11 and 12; Sarjoo 52-*qGN4.1* has a large 8.7 Mb DP segment on chromosome 4 and 50 smaller segments of >0.2 Mb on chromosomes 2, 9, 11 and 12; Pusa 44-*qGN4.1* has 104 DP segments on chromosomes 1, 2, 3, 5, 11 and 12; CSR 30-*qGN4.1* has three large DP segments on chromosomes 3 (1.4 Mb), 5 (0.3 Mb) and 8 (2.4 Mb); Ranjit-*qGN4.1* has a large DP segment on chromosome 1(1.1 Mb), four separate large segments of >0.2 Mb on chromosome 3, and 0.2 Mb on chromosome 8; CR 1009-*qGN4.1* has two large DP segments 1.1 Mb and 1.9 Mb on chromosome 1, 0.9 Mb on chromosome 3; 1.0 Mb and 0.9 Mb on chromosome 6, 1.4 Mb on chromosome 9 and 1.1 Mb on chromosome 12; and Pusa Basmati 1-*qGN4.1* has 85 DP segments of 0.2 Mb on chromosomes 2, 3, 6, 8, 9,10, 11 and 12. Thus, the high-resolution background analysis using 50 K SNP chip showed high RPG recovery in Pusa Basmati 1121, IR 64, CSR 30, Ranjit and CR 1009, and need for additional back crossing in Samba Mahsuri, Swarna, MTU 1010, HUR 105, Sarjoo 51, Pusa 44 and Pusa Basmati 1 for the elimination of the remaining large DP chromosome segments to develop QTL-NILs with uniform high RPG recovery.

**Fig. 6:**
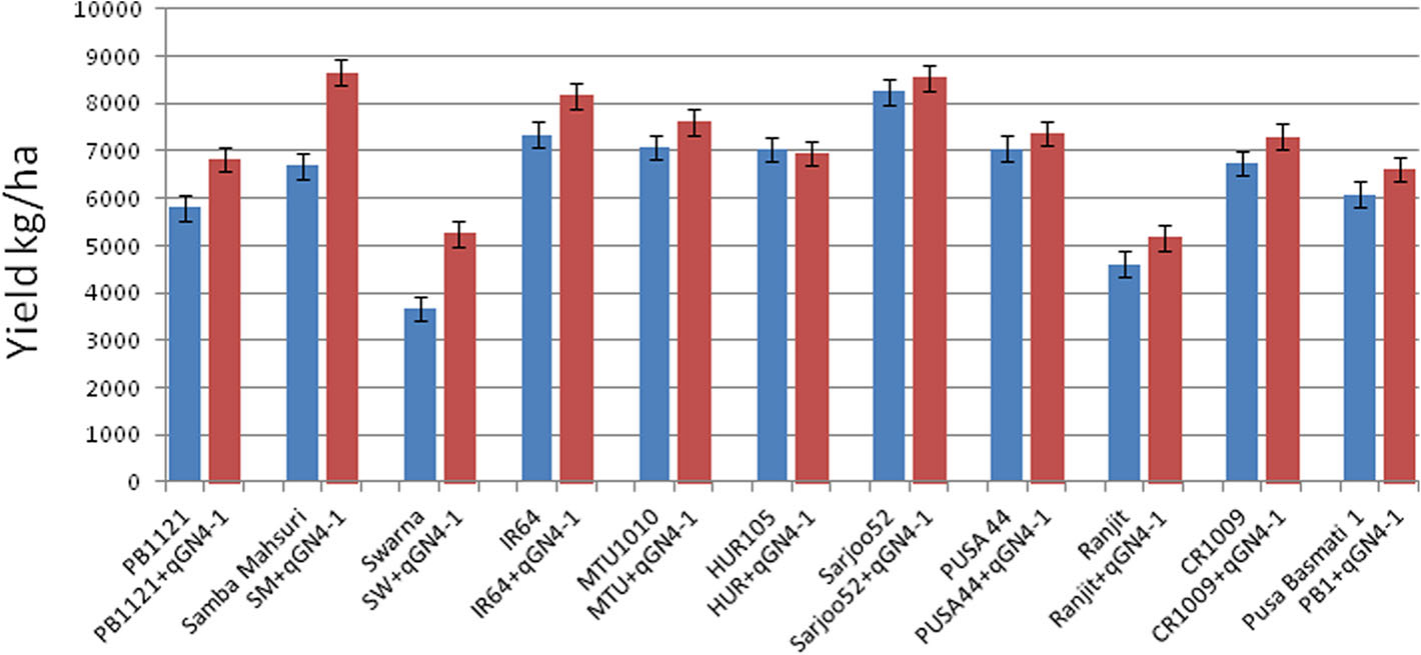
Bar diagram showing yield performance of *qGN4.*1 QTL-NILs in 11 different backgrounds of rice in comparison to their recipient parents. Values are average of two replications, with the whiskers showing S.E. of means. SM = Samba Mahsuri; SW = Swarna

The QTL-NILs with the highest RPG recovery in 11 different varietal backgrounds were evaluated for yield performance in large replicated field plots. Yield data could not be obtained for CSR 30 because of poor seed setting due to environmental factors at late maturity. There was a significant increase in yield with standard agronomic practices with the introgression of *qGN4.1* QTL in PB 1121, Samba Mahsuri and Swarna backgrounds as compared to their respective RPs, but the maximum gain was observed in Samba Mahsuri genetic background where the yield was enhanced from 6690 kg/ha in the RP Samba Mahsuri to 8667 kg/ha in Samba Mahsuri-*qGN4.1*. Significant yield increases were also observed with Pusa Basmati 1121-*qGN4.1* from 5808 kg/ha for RP to 6833 kg/ha for QTL-NIL, and with Swarna-*qGN4.1* from 3667 kg/ha for RP to 5250 kg/ha for QTL-NIL. Other eight varieties also showed the numerical superiority of yield for *qGN4.1* QTL-NILs as compared to the RP control, but the gain was not statistically significant (Fig. 6; Additional file 7: Table S4). After further multilocation evaluation of yield performance, some of these QTL-NILs have the potential to be released for commercial cultivation because of high acceptability of the RP varieties.

### Differential Expression of *Nal1* and Other Candidate Genes in the *qGN4.1* QTL Interval

The *qGN4.1* QTL in the marker interval nkssr 04-02 and nkssr 04-19 (11.1 cM, 0.78 Mb) was identified as the most important region controlling the grain number per panicle in the NPT genotype Pusa 1266 (Deshmukh et al. 2010, Marathi et al. 2012). Other studies have also shown the involvement of this chromosomal region on photosynthetic efficiency and plant architecture including the number of spikelets per panicle (Takai et al. 2013; Fujita et al. 2013; Zhang et al. 2014). This region has the evidence for the presence of 148 expressed genes in the EST database (TIGR Version 7.0, http://rice.plantbiology.msu.edu/). This list was too large for identification of causal gene(s) underlying *qGN4.1* QTL. Therefore, QTL mapping was complemented with genome-wide transcriptome profiling to identify differentially expressed genes at early panicle development stage (Deshmukh et al. 2010). Microarray-based transcriptome profiling revealed eight genes in the *qGN4.1* region that were differentially expressed between the two parents, including *Nal1* gene (LOC_Os04g52479) that codes for a serine and cysteine protease. Of the eight differentially genes, only one coding for protein kinase domain-containing protein (LOC_Os04g52590) showed overexpression in the high grain number genotype; *Nal1* gene was actually downregulated by −4.0 and −1.1 folds in microarray and qRT-PCR studies, respectively (Deshmukh et al. 2010). Hence, we analyzed differential expression of these two potential candidate genes along with a third neutral gene coding for retrotransposon protein (LOC_Os04g52540) in all the 12 QTL-NILs developed in the present study. Consistent with our earlier results (Deshmukh et al. 2010), the LOC_Os04g52590 gene coding for protein kinase domain-containing protein was consistently overexpressed in QTL-NILs of all the 12 RP backgrounds (Table 3). Also, consistent with our earlier results (Deshmukh et al. 2010), *Nal1* (LOC_Os04g52479) and retrotransposon (LOC_Os04g52540) genes actually showed down regulation in the *qGN4.1* QTL-NILs (Table 3). This is different from the situation with *SPIKE*, *GPS* and *LSCHL4* QTLs where overexpression of *Nal1* gene is shown to be the causal factor for high spikelet number (Fujita et al. 2013; Takai et al. 2013; Zhang et al. 2014). The role of overexpressed gene (LOC_Os04g52590) coding for protein kinase domain-containing protein in *qGN4.1* QTL-NILs is being further investigated by genetic recombination and transformation studies.

**Table 3:** Fold Change in the Expression Level of Three Genes Located in the *qGN4.1* QTL Interval in the Panicle Primordia of Twelve QTL Introgression Lines in Comparison to the Respective Recipient Parents as Detrmined Using qRT-PCR

Genotype	Gene Id.		
	LOC Os04g52479	LOC_ Os04g52540	LOC Os04g52590
PB1121 + *qGN4.1*	−0.1	−0.9*	+4.2**
Samba Mahsuri + *qGN4.1*	−0.7*	−0.1	+2.2**
Swarna + *qGN4.1*	−0.7 *	−0.8*	+1.9**
IR64 + *qGN4.1*	0.6 *	−0.3	+2.1**
MTU1010 + *qGN4.1*	0.5*	−0.6*	+1.7**
HUR105 + *qGN4.1*	0.1	−0.4	+0.9**
Sarjoo52 + *qGN4.1*	−0.3	−0.2	+3.2**
PUSA44 + *qGN4.1*	−0.4	−0.2	+1.6**
CSR30 + *qGN4.1*	−0.3	−0.3	+0.9**
Ranjit + *qGN4.1*	−0.6 *	−0.5*	+2.3**
CR1009 + *qGN4.1*	−0.5*	−0.8*	+2.6 **
PB1 + *qGN4.1*	−0.5*	−0.5*	+2.2**

Significant at *P < 0.01, **P < 0.001 by t test

## Conclusions

This study describes the development and evaluation of NILs with *qGN4.1* QTL for high grain number per panicle in the backgrounds of 12 different mega varieties of rice. Introgression of *qGN4.1* resulted in a significant increase in the number of grains per panicle in all the 12 backgrounds. The associated morphological traits such as flag leaf width and the number of primary and secondary branches in the panicle also increased significantly, but the total number of tillers was reduced. However, the number of productive tillers remained unchanged in all backgrounds except Pusa Basmati 1121 and Sarjoo 52, which showed a significant reduction in the number of productive tillers per plant. Even with the normal planting density, the QTL-NILs showed significantly higher yield as compared to the respective RPs in the field experiments for three varieties, namely Pusa Basmati 1121, Samba Mahsuri and Swarna. Our results showed that the *qGN4.1* QTL can be effectively deployed to further enhance the yield potential of popular mega varieties of rice.

## Additional file

Additional file 1: Table S6. The sequence of DNA marker and its physical position using pseudomolecule release 7 of TIGR on chromosome 4 used for introgression of QTL *qGN4.1* into 12 different mega rice varieties. (DOCX 11 kb)
Additional file 2: Table S1. Number of plants after foreground and recombinant selection for *qGN4*.*1* QTL for high grain number into 12 different mega varieties of rice at different backcross generations. (DOCX 34 kb)
Additional file 3: Table S2. Adjusted means for different morphological agronomic traits of the six different *qGN4.1* QTL-NILs for each variety along with respective recipient parents in BC2F3 generation. (XLSX 23 kb)
Additional file 4: Figure S1. Plant architecture of ***qGN4.1*** QTL-NILs (left side) of rice as compared to their recipient parents (right side): (D) IR 64 (E) MTU 1010 (F) HUR 105 (scale bars: 10 cm). (TIFF 4119 kb)
Additional file 5: Figure S2. Plant architecture of ***qGN4.1*** QTL-NILs (left side) of rice as compared to their recipient parents (right side): (G) Sarjoo 52 (H) Pusa 44 (I) CSR 30 (scale bars: 10 cm). (TIFF 3856 kb)
Additional file 6: Figure S3. Plant architecture of ***qGN4.1*** QTL-NILs (left side) of rice as compared to their recipient parents (right side): (J) Ranjit (K) CR 1009 (L) Pusa Basmati 1(PB 1) (scale bars: 10 cm). (TIFF 4098 kb)
Additional file 7: Table S3. Percentage genome similarity of the best eight *qGN4.1* QTL-NILs of Samba Mahsuri, Swarna, MTU 1010, Sarjoo 52, Pusa 44, Pusa Basmati 1 and best two lines of Pusa Basmati 1121, IR 64, HUR 105, CSR 30, Ranjit, CR 1009 with their respective recipient parent at BC3F2 generation in the genetic background of rice analysed using OsSNPnks SNP genotyping chip. (DOCX 33 kb)
Additional file 8: Figure S4. Graphical representation of RPG similarity with *qGN4.1* QTL-NILs of (A) Samba Mahsuri, (B) Swarna (C) IR 64 (D) MTU 1010 (E) HUR 105(F) Sarjoo 52 (Blue colors denotes recipient segment, Red denotes donor segment and Green denotes Heterozygote). (TIFF 558 kb)
Additional file 9: Figure S5. Graphical representation of RPG similarity with *qGN4.1* QTL-NILs of (G) Pusa 44 (H) CSR 30(I) Ranjit (J) CR 1009 (K) Pusa Basmati 1(PB 1) (Blue colors denotes recipient segment, Red denotes donor segment and Green denotes Heterozygotes). (TIFF 476 kb)
Additional file 10: Table S5. Analysis of all the affymetrix 50 K SNP calls of 12 RPG along with *qGN4.1* QTL-NILs on separate sheets. (XLSX 99942 kb)
Additional file 11: Table S4. Yield performance of *qGN4.1* QTL-NILs in 11 different backgrounds in comparison to their recipient parents (data not available for CSR 30). Values are average of two replications, with LSD at 5%. SM = Samba Mahsuri; SW = Swarna. (DOCX 14 kb)
Additional file 12: Table S7. Number of donor parent genome segments of larger than 0.2 Mb in the *qGN4.1*-NILs in recipient genetic backgrounds of 12 different mega varieties of rice. (XLSX 11 kb)

## Abbreviations

BC: Backcross
CD: Critical difference
DP: Donor parent
MABB: Marker-assisted backcross breeding
NIL: Near-isogenic line
NPT: New pant type
qRT-PCR: Quantitative Reverse transcriptase-polymerase chain reaction
QTL: Quantitative trait locus
RIL: Recombinant inbred line
RP: Recipient parent
RPG: Recipient parent genome
SNP: Single nucleotide polymorphism
SSR: Simple sequence repeat
