# Supplementary material for: Effect of qGN4.1 QTL for Grain Number per Panicle in Genetic Backgrounds of Twelve Different Mega Varieties of Rice
Source: Rice (N Y). 2018 Jan 22;11:8. doi: 10.1186/s12284-017-0195-9 (PMC5777967; doi:10.1186/s12284-017-0195-9)
Supplement: Additional file 1: Table S6. — The sequence of DNA marker and its physical position using pseudomolecule release 7 of TIGR on chromosome 4 used for introgression of QTL qGN4.1 into 12 different mega rice varieties. (DOCX 11 kb) [file 12284_2017_195_MOESM1_ESM.docx]

| S.No. | Primer_Id | Position* | Left primer sequence | Right primer sequence |
| --- | --- | --- | --- | --- |
| 1 | RM2441 | 28046537 | ATTAACAGATGATGCAAATC | CCATGTGAGTTTAAATTCAC |
| 2 | NKSSR04-11 | 30903920 | CCATCAGTTGAAGGGCTCTC | CTTTTATGGCATGGGCAACT |
| 3 | NKSSR04-19 | 31262441 | CTGGAATCACAAACCACGAC | GCTACCTCAAGCTCCACGAC |
| 4 | HvSSR04-49 | 33488512 | ATAGCTCAAGTGCAAGAACC | AAATTTGATGTACCGGCTAA |

*Position on rice chromosome 4 pseudomolecule release 7 of TIGR
