# Supplementary figures and images for: Effect of qGN4.1 QTL for Grain Number per Panicle in Genetic Backgrounds of Twelve Different Mega Varieties of Rice
Source: Rice (N Y). 2018 Jan 22;11:8. doi: 10.1186/s12284-017-0195-9 (PMC5777967; doi:10.1186/s12284-017-0195-9)

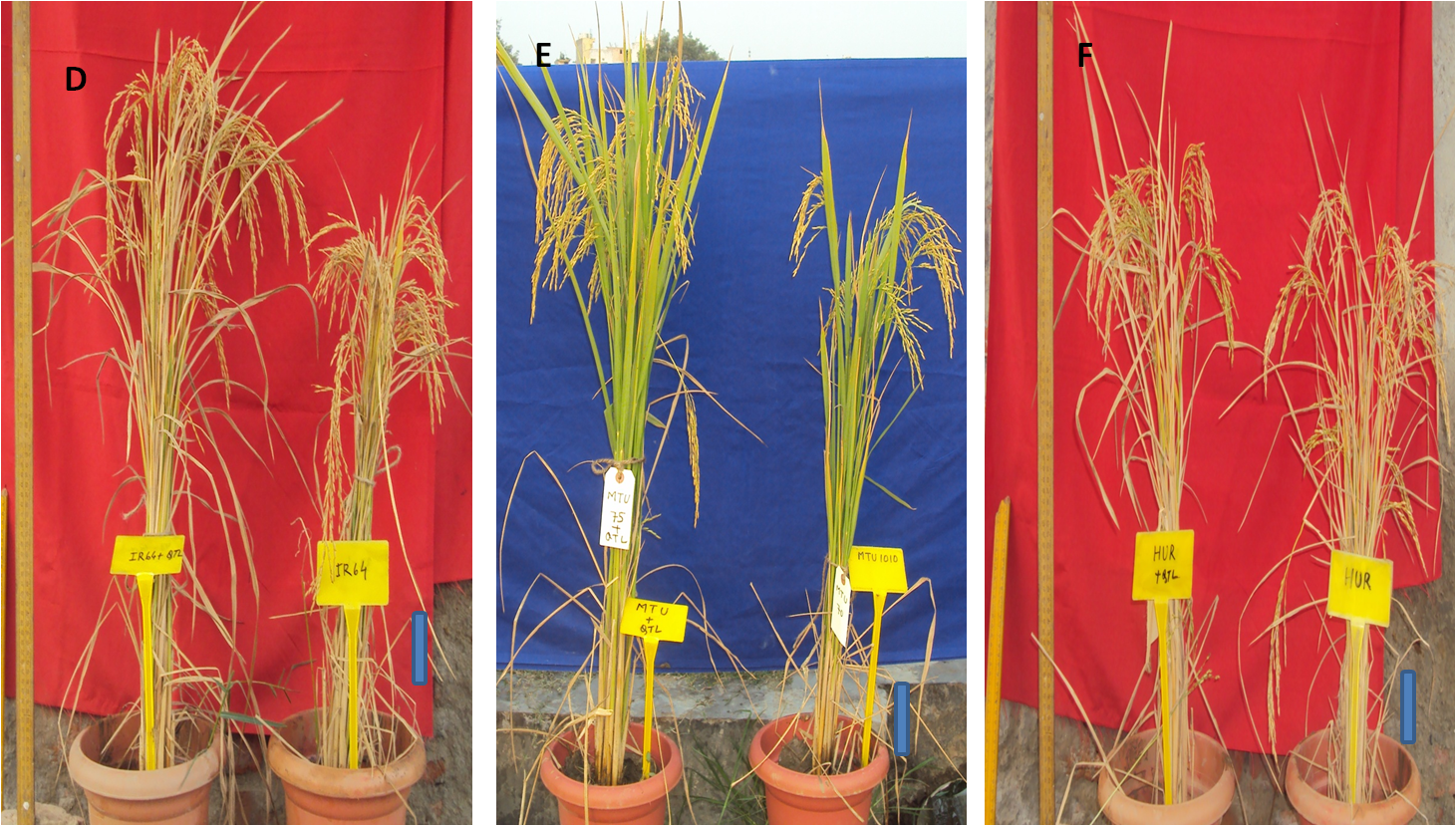

Supplement: Additional file 4: Figure S1. — Plant architecture of qGN4.1 QTL-NILs (left side) of rice as compared to their recipient parents (right side): (D) IR 64 (E) MTU 1010 (F) HUR 105 (scale bars: 10 cm). (TIFF 4119 kb) [file 12284_2017_195_MOESM4_ESM.tif]

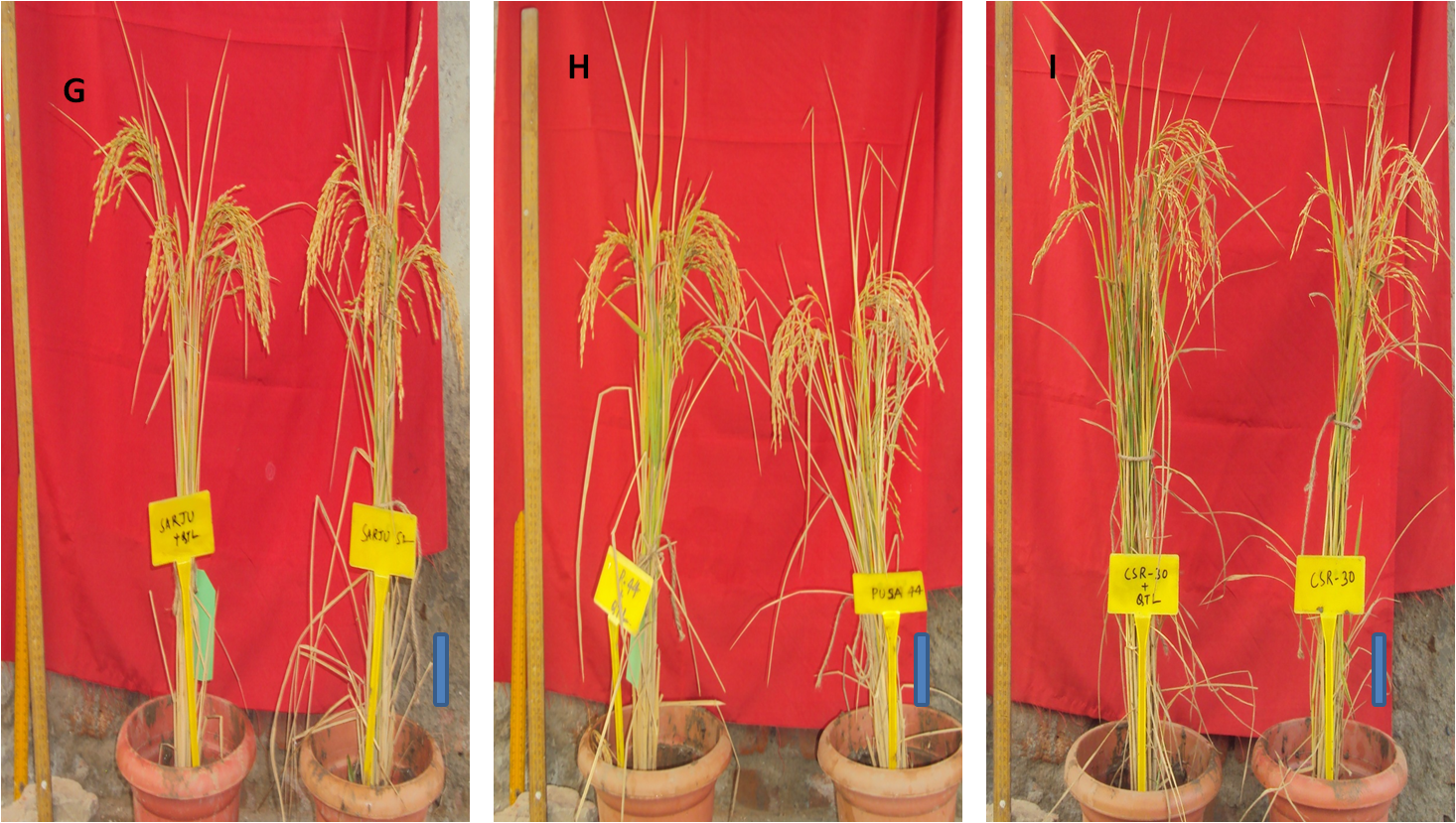

Supplement: Additional file 5: Figure S2. — Plant architecture of qGN4.1 QTL-NILs (left side) of rice as compared to their recipient parents (right side): (G) Sarjoo 52 (H) Pusa 44 (I) CSR 30 (scale bars: 10 cm). (TIFF 3856 kb) [file 12284_2017_195_MOESM5_ESM.tif]

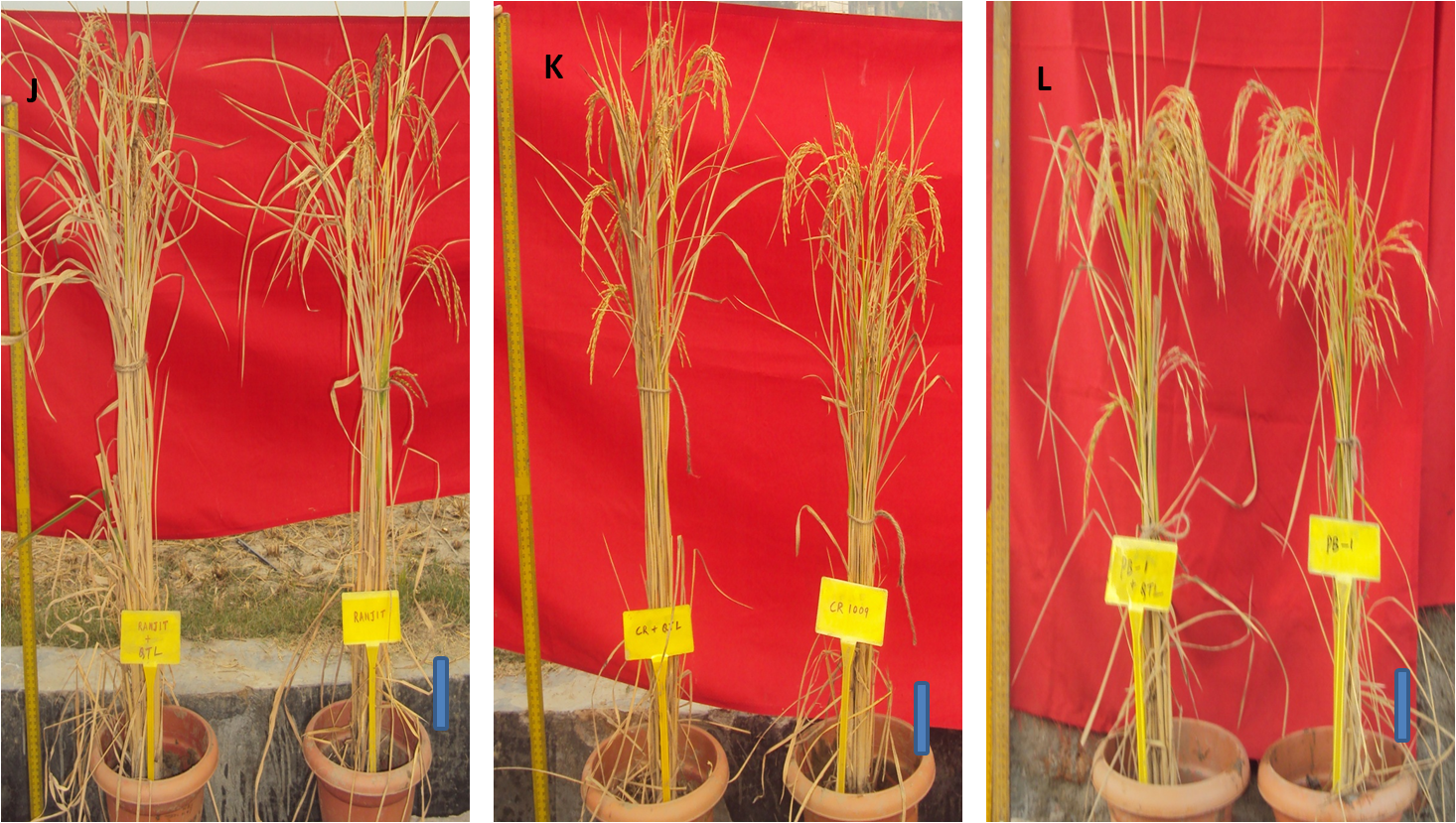

Supplement: Additional file 6: Figure S3. — Plant architecture of qGN4.1 QTL-NILs (left side) of rice as compared to their recipient parents (right side): (J) Ranjit (K) CR 1009 (L) Pusa Basmati 1(PB 1) (scale bars: 10 cm). (TIFF 4098 kb) [file 12284_2017_195_MOESM6_ESM.tif]

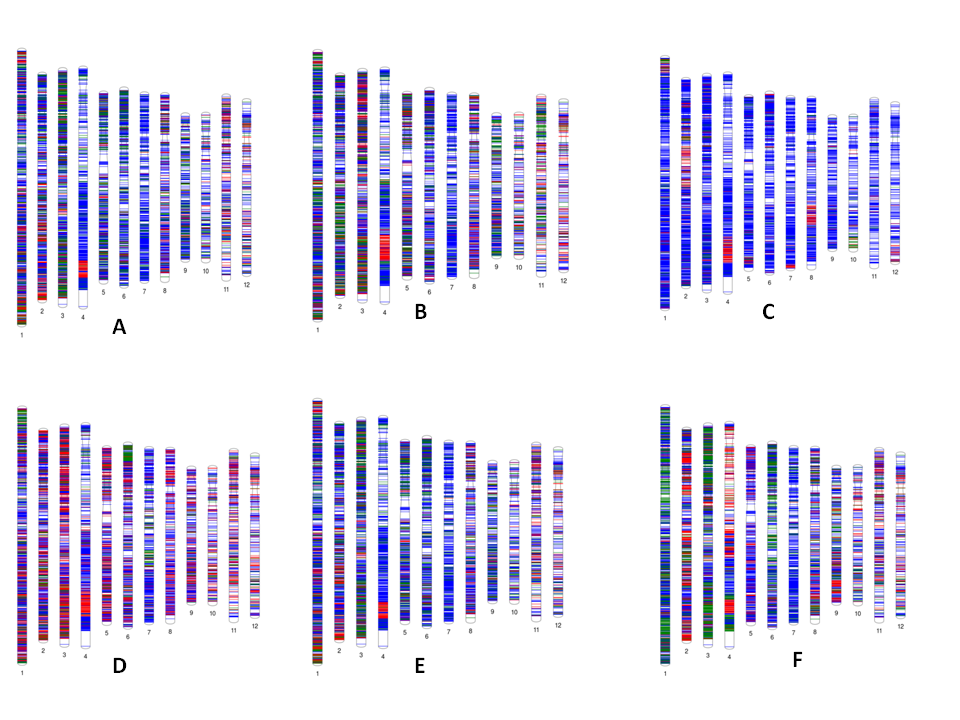

Supplement: Additional file 8: Figure S4. — Graphical representation of RPG similarity with qGN4.1 QTL-NILs of (A) Samba Mahsuri, (B) Swarna (C) IR 64 (D) MTU 1010 (E) HUR 105(F) Sarjoo 52 (Blue colors denotes recipient segment, Red denotes donor segment and Green denotes Heterozygote). (TIFF 558 kb) [file 12284_2017_195_MOESM8_ESM.tif]

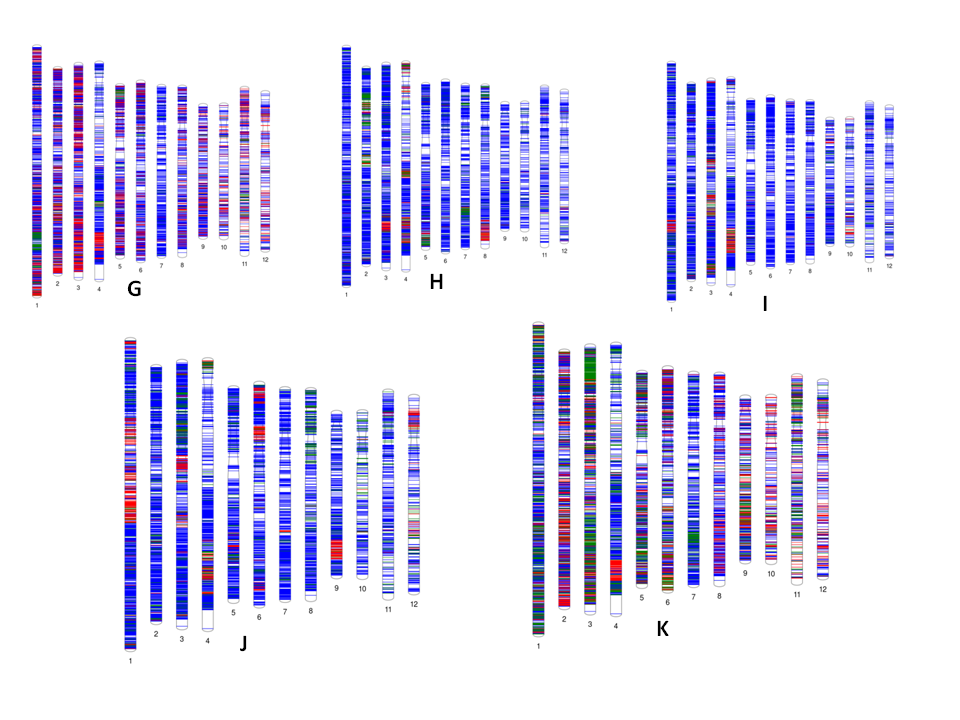

Supplement: Additional file 9: Figure S5. — Graphical representation of RPG similarity with qGN4.1 QTL-NILs of (G) Pusa 44 (H) CSR 30(I) Ranjit (J) CR 1009 (K) Pusa Basmati 1(PB 1) (Blue colors denotes recipient segment, Red denotes donor segment and Green denotes Heterozygotes). (TIFF 476 kb) [file 12284_2017_195_MOESM9_ESM.tif]
