# Supplementary material for: Effect of qGN4.1 QTL for Grain Number per Panicle in Genetic Backgrounds of Twelve Different Mega Varieties of Rice
Source: Rice (N Y). 2018 Jan 22;11:8. doi: 10.1186/s12284-017-0195-9 (PMC5777967; doi:10.1186/s12284-017-0195-9)
Supplement: Additional file 7: Table S3. — Percentage genome similarity of the best eight qGN4.1 QTL-NILs of Samba Mahsuri, Swarna, MTU 1010, Sarjoo 52, Pusa 44, Pusa Basmati 1 and best two lines of Pusa Basmati 1121, IR 64, HUR 105, CSR 30, Ranjit, CR 1009 with their respective recipient parent at BC3F2 generation in the genetic background of rice analysed using OsSNPnks SNP genotyping chip. (DOCX 33 kb) [file 12284_2017_195_MOESM7_ESM.docx]

| Variety | Percent Recovery in *qGN4.1* QTL NILs | | | | | | | |
| --- | --- | --- | --- | --- | --- | --- | --- | --- |
|  | 1 | 2 | 3 | 4 | 5 | 6 | 7 | 8 |
| Samba Mahsuri | 96.5 | 94.9 | 95.1 | 95.2 | 95.5 | 95.0 | 93.6 | 94.5 |
| Swarna | 92.9 | 92.7 | 91.9 | 92.7 | - | - | 92.6 | 93.8 |
| MTU 1010 | 91.3 | 92.5 | 93.6 | 93.2 | 92.3 | 93.6 | 92.8 | 93.8 |
| Sarjoo 52 | 80.0 | 90.7 | - | - | 89.2 | 90.0 | 92.0 | 91.0 |
| Pusa 44 | 92.0 | 92.2 | 91.3 | 94.3 | 92.7 | 91.6 | 91.8 | 90.9 |
| PB 1 | 93.4 | 91.8 | 91.3 | 93.0 | 89.9 | 92.3 | 91.7 | 92.6 |
| PB 1121 | 97.6 | 97.5 |  |  |  |  |  |  |
| IR 64 | 96.8 | 98.0 |  |  |  |  |  |  |
| HUR 105 | 96.4 | 96.5 |  |  |  |  |  |  |
| CSR 30 | 97.4 | 97.2 |  |  |  |  |  |  |
| Ranjit | 97.0 | 97.7 |  |  |  |  |  |  |
| CR 1009 | 96.0 | 95.9 |  |  |  |  |  |  |
