# Supplementary material for: Effect of qGN4.1 QTL for Grain Number per Panicle in Genetic Backgrounds of Twelve Different Mega Varieties of Rice
Source: Rice (N Y). 2018 Jan 22;11:8. doi: 10.1186/s12284-017-0195-9 (PMC5777967; doi:10.1186/s12284-017-0195-9)
Supplement: Additional file 11: Table S4. — Yield performance of qGN4.1 QTL-NILs in 11 different backgrounds in comparison to their recipient parents (data not available for CSR 30). Values are average of two replications, with LSD at 5%. SM = Samba Mahsuri; SW = Swarna. (DOCX 14 kb) [file 12284_2017_195_MOESM11_ESM.docx]

| Variety | Yield kg/ha |
| --- | --- |
| Pusa Basmati 1121 (PB1121) | 5808.3 |
| PB1121+*qGN4.1* | 6833.3* |
| Samba Mahsuri | 6690.0 |
| Samba Mahsuri+*qGN4.1* | 8666.7* |
| Swarna | 3666.5 |
| Swarna+*qGN4.1* | 5249.8* |
| IR 64 | 7354.2 |
| IR64+*qGN4.1* | 8166.7 |
| MTU1010 | 7083.3 |
| MTU1010+*qGN4.1* | 7625.0 |
| HUR 105 | 6958.3 |
| HUR105+*qGN4.1* | 7041.7 |
| Sarjoo 52 | 8250.0 |
| Sarjoo52+*qGN4.1* | 8541.7 |
| PUSA 44 | 7055.8 |
| PUSA44+*qGN4.1* | 7375.0 |
| Ranjit | 4604.2 |
| Ranjit+*qGN4.1* | 5166.7 |
| CR 1009 | 6750.0 |
| CR1009+*qGN4.1* | 7308.3 |
| Pusa Basmati 1(PB 1) | 6083.3 |
| PB1+*qGN4.1* | 6625.0 |
| LSD at 5% P | 990.9 |
